# Thermal-Assisted Field Emission Characteristics of Carbon Nanotubes and Application in Pulsed X-Ray Imaging

**DOI:** 10.3390/nano16050282

**Published:** 2026-02-24

**Authors:** Zhiqiang Xia, Shichao Feng, Xiaodong Sun, Chi Li, Zhenjun Li, Liye Zhao

**Affiliations:** 1 Key Laboratory of Micro-Inertial Instrument and Advanced Navigation Technology, Ministry of Education, School of Instrument Science and Engineering, Southeast University, Nanjing 210096, China; 2 Laboratory of Nanophotonic Materials and Devices, Laboratory of Standardization and Measurement for Nanotechnology, National Center for Nanoscience and Technology, Beijing 100190, China; 3 China Ship Scientific Research Center, Wuxi 214082, China

**Keywords:** carbon nanotube, field emission, X-ray imaging, pulse response

## Abstract

Carbon nanotube (CNT) cathode materials exhibit excellent electron emission performance and have become a key research focus in the field of vacuum electronics. However, their practical applications are still restricted by challenges, including emission instability and ambiguity in temporal resolution capability. This work investigated the thermal-assisted field emission characteristics of CNT and their application in pulsed X-ray imaging. Systematic characterization of the turn-on field strength, emission stability, pulse response characteristics, and pulsed X-ray imaging performance demonstrated that the thermal-assisted operating mode reduced current fluctuations to below 1%. Increasing the heating power further enhanced emission stability and lowered the turn-on field strength. In thermal-assisted pulsed emission mode, CNT cathodes exhibited reduced power consumption compared to conventional thermionic cathodes and achieved microsecond-scale pulse response. Further X-ray imaging experiments confirmed that the X-ray dose generated by CNT in this operational mode exhibited higher stability, enabling 100 μs pulsed imaging and clear visualization of rotating blades operating at 600 Hz. This study validated the feasibility of CNT cathodes for high-speed X-ray imaging and could provide a reference for the development of advanced pulsed X-ray sources and related technologies.

## 1. Introduction

The electron source serves as the core component of vacuum electronic devices. Early electron microscopes and vacuum tubes mainly relied on thermionic cathodes, which emit electrons via thermal excitation of the cathode material. Thermionic cathodes are known for their stable emission characteristics and have been extensively studied for more than a century [1]. However, they require operational temperatures as high as 1200 °C, resulting in slow response times and significant thermal load. Since the late twentieth century, field emission cold cathodes such as CNTs have attracted considerable interest due to their fast response, low power consumption, and high current density [2,3,4,5,6,7]. Nevertheless, high surface local electric field intensity, along with a tendency to adsorb gas molecules and susceptibility to ion bombardment, leads to issues such as large fluctuations in emission current and cathode degradation [8,9], which restrict their applications in vacuum electronic devices.

Recent studies have demonstrated that the electron emission performance of CNTs is significantly enhanced by the application of heat, whether applied as a pretreatment prior to field emission or as thermal assistance during the emission process [10,11]. The observed improvements include greater emission stability and a reduction in the turn-on field strength. Extensive research has also focused on introducing thermal assistance to CNT field emitters for improved field emission performance. Research teams from South Korea had attempted to improve the emission performance of CNT via substrate heating or high-temperature annealing. For example, Yuning Sun et al. reduced the turn-on field strength of CNT films from 1.62 V/μm to 1.15 V/μm by annealing the films at 900 °C [12]. Syed Muhammad Zain Mehdi and co-workers enhanced the density of effective field emission sites on the surface of CNT films via thermal purification. This treatment endowed high-purity crystalline arc discharge multi-walled carbon nanotubes (AD-MWCNTs) with exceptional field emission properties. Highly pure crystalline AD-MWCNTs exhibited high-performance field-emission properties, namely a high current density of 2.7 A/cm^2^ and long-term emission stability (12 h at a current density of 500 mA/cm^2^), with no performance reduction [13]. Guo Dabo’s team reported progress on thermo-field synergy [14]. The influence of temperature variation on the field emission properties of CNTs deposited on tungsten wire tips was examined, and it was observed that as the temperature increased from 50 °C to 150 °C, the turn-on field strength of the CNT-based field emission decreased from 2.3 MV/m to 2.0 MV/m. However, most investigations focusing on thermal treatment and thermal assistance remain primarily confined to optimizing field-emission performance parameters, with relatively insufficient research on their practical implementation in functional devices. In fact, the enhancements mentioned in emission performance are of critical significance for CNTs to serve as cold-cathode electron sources, enabling efficient and stable X-ray output [15].

Therefore, this work systematically tested the turn-on field strength, emission stability, transient response and X-ray imaging performance of CNT cold cathodes under thermal-assisted mode. The results validate the feasibility of this approach for high-speed X-ray imaging, providing a reference for advancing next-generation pulsed X-ray detection technologies.

## 2. Experimental Section

### 2.1. Preparation and Characterization of CNT Cathodes

**Figure A1:**
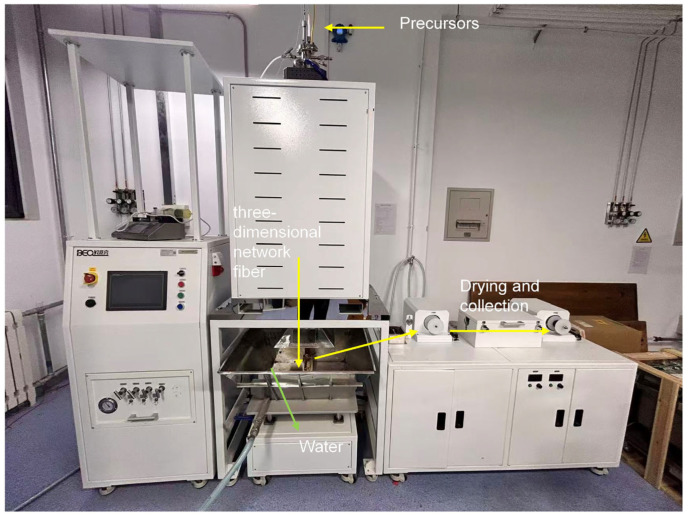
Schematic of the CNT growth process.

CNTs were prepared by chemical vapor deposition (CVD) in this study. Ferrocene (FeCp_2_) (Damao Chemical Reagent Factory, Tianjin, China) was used as the catalyst, and anhydrous ethanol (Sinopharm Chemical Reagent Co., Ltd., Shanghai, China) served as both the carbon source and solvent. A precursor solution was prepared at a defined stoichiometric ratio, subjected to ultrasonic treatment, and heated to form a homogeneous and stable mixture. The reaction zone was then heated to 1200 °C, while a high-purity Ar/H_2_ mixture was continuously introduced as the carrier and reducing gas. An atomized injection system was employed to feed the precursor into the high-temperature zone at a constant rate. Under these conditions, FeCp_2_ decomposed to form nanoscale Fe catalyst particles, and ethanol pyrolyzed to generate carbon species. Carbon atoms subsequently precipitated and self-assembled on the catalyst surface, resulting in CNTs with a three-dimensional network structure. After being compressed into bundles in a water bath with a depth of 0.2 m and a pressure of ~1960 Pa, the CNTs were dried at 150 °C and collected at a speed of 20 mm/min on a reel (Figure A1). This process yielded dense CNT bundles with low impurity content, which were used as electron-emitting cathodes.

The morphology of the CNT samples was examined using a Hitachi S-4800 scanning electron microscope (Hitachi High-Technologies Corporation, Tokyo, Japan). Raman spectra of the cathode tips were collected with a Renishaw inVia plus laser Raman spectrometer (Renishaw plc, Wotton-under-Edge, Gloucestershire, UK) to assess their structural characteristics and degree of defect. Both SEM and Raman analyses were conducted before and after the emission experiments to investigate the influence of thermal assistance on the field emission behavior of CNTs.

### 2.2. Continuous and Pulsed Field-Emission Testing

**Figure 1:**
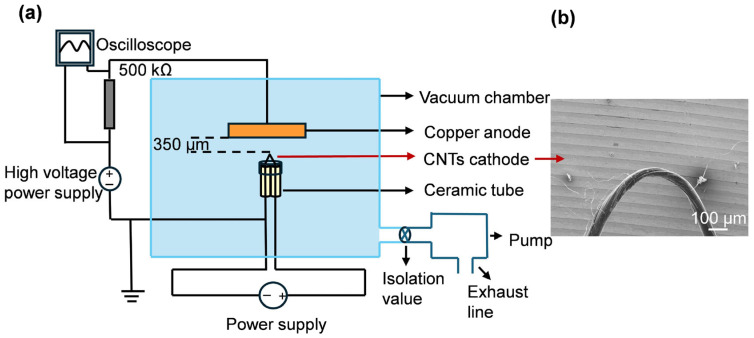
Schematic diagram of the thermal-assisted field emission experimental setup for CNTs. (**a**) Schematic diagram of the experimental setup. (**b**) SEM image of the CNT bundle cathode.

The CNT bundle was folded into a V-shaped configuration, with two protruding pins guided through two ceramic tubes on either side. The pins were bonded to copper wires using silver conductive epoxy adhesive. The copper wires were then used to extend the connections beyond the ceramic tubes and were connected to the positive and negative terminals of a low-voltage power supply, respectively. A pure copper block was used as the anode plate and connected to a positive high-voltage power supply. The distance between the cathode tip and the anode was approximately 350 μm. A voltage-dividing resistor of 500 kΩ was incorporated at the anode side during the field emission measurement. The heating power was adjusted using a variable power supply. (Figure 1a) Tests were conducted under heating powers of 0, 0.5, 1.0, 1.5, and 2.0 W. The corresponding current values were 0, 0.143 A, 0.19 A, 0.225 A, and 0.254 A. Due to the experimental configuration, distinct heating conditions in subsequent analyses are differentiated by heating power.

For the pulsed emission testing, which was conducted under identical conditions, the resistor was replaced with one of 14.2 kΩ to reduce the circuit impedance and adjust the voltage division. The voltages across both the resistor and the pulsed power supply were recorded using an DSOX6004A oscilloscope(Keysight Technologies Microwave Products (M) Sdn. Bhd., Pulau Pinang, Malaysia). Furthermore, a comparative experiment was conducted using tungsten wire, which was a conventional X-ray source, to examine the differences in emission current between the two materials under identical thermal-assisted conditions and to evaluate the influence of heating power variation on their pulse responses. The performance of both CNTs and tungsten wire as cathode materials was systematically analyzed, providing a technical basis for subsequent X-ray imaging experiments.

### 2.3. X-Ray Imaging Experiments

A typical X-ray imaging configuration was employed, using the project-developed CNT-based X-ray source, a high-speed rotating blade, and an PaxScan 1313DX X-ray detector(Varian Medical Systems, Palo Alto, CA, USA) to perform absorption-based radiography. The electron emission device employs a two-electrode configuration, consisting of a Joule-heated CNT cathode and a gate electrode. The intensity of X-rays generated under varied heating conditions and the temporal response of pulsed X-rays were recorded, with the chamber vacuum maintained at ~5 × 10^−6^ Pa.

**Figure A2:**
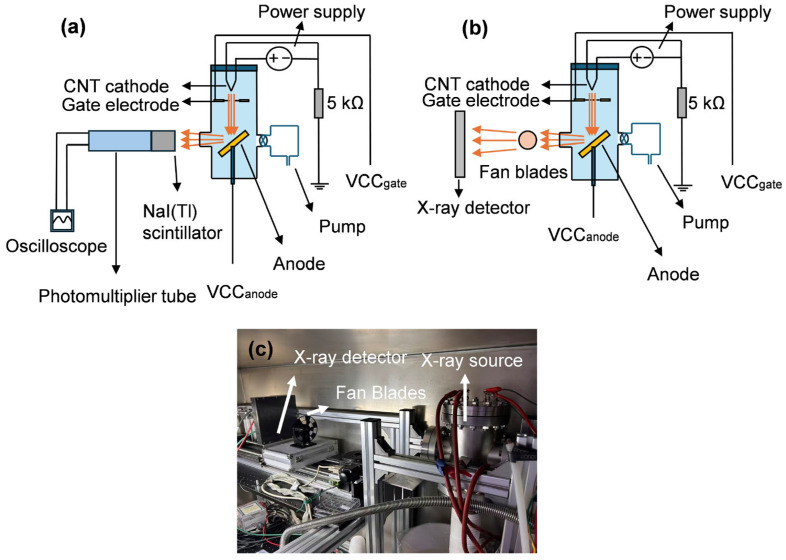
Schematic of the X-ray application experimental setup. (**a**) Schematic diagram of continuous X-ray performance testing. (**b**) Schematic of the pulsed X-ray imaging setup. (**c**) Photographic image of the pulsed X-ray imaging apparatus.

Based on the principle where thallium-doped sodium iodide crystals convert X-rays into visible light, X-ray intensity was sampled at 0.5 h intervals over a 24 h period using photomultiplier tubes. The signals detected by the tubes were adjusted by a gain amplifier and recorded with a multi-channel oscilloscope. The testing setup for continuous light emission performance is shown in Figure A2a. In pulse testing, the correlation between pulse voltage and X-ray response was recorded using an oscilloscope. For imaging applications, an adjustable-speed fan blade was selected as the test object, and the imaging results were collected through an X-ray detector (Figure A2b,c). With the detector’s maximum frame rate set at 30 fps, the pulse voltage width was configured to 100 μs at a frequency of 30 Hz. Since the X-ray pulse duration was significantly shorter than the detector’s integration time per frame, single-pulse exposure imaging was achieved. The rotational frequency of fan blades was sequentially set to 1, 10, 20, and 30 Hz, and was further increased to 600 Hz to evaluate the maximum imaging capability in pulse mode.

## 3. Results and Discussion

### 3.1. Field Emission Performance Testing and Analysis of CNT Cathode

**Figure 2:**
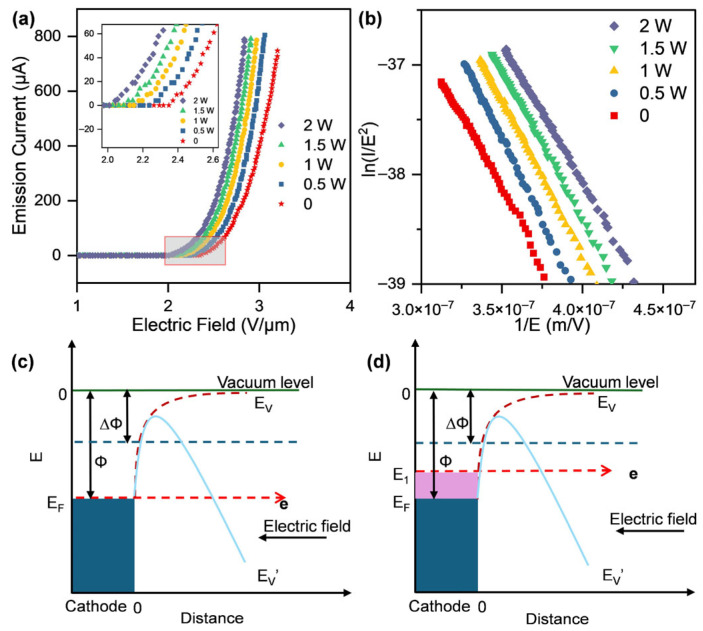
Performance and mechanism of thermal-assisted field emission from CNTs. (**a**) Current vs. electric field intensity curves of CNT cathode at heating powers ranging from 0 to 2 W. (**b**) The M-G curve corresponding to the I-E curve. (**c**) Electron emission mechanism with only an external electric field applied. (**d**) Electron emission mechanism with application of thermal assistance and an external electric field.

Smooth looped fiber sidewalls demonstrate limited field emission capability [16]. To further understand the electron emission behavior at the tip of the V-shaped CNT cathode, field emission characterization was performed under varying heating powers with a direct current (DC) voltage applied to the anode. The results are shown in Figure 2. The cathode current increases exponentially with the applied field, with turn-on field intensities of 2.371, 2.274, 2.177, 2.117, and 2.043 V μm^−1^ (Figure 2a). This indicates a decrease in the turn-on field as heating power increases. To provide a quantitative interpretation of the measured E–I characteristics under thermal assistance, the experimental data were analyzed within the framework of the Murphy–Good (MG) field emission theory [17]. Figure 2b presents the corresponding Murphy–Good plots [18], which maintain excellent linearity across all experimental conditions. The slopes derived from linear fitting are −34,278,399.56, −34,708,626.21, −32,959,011.71, −31,281,058.31, and −29,062,327.87, respectively, for the increasing heating powers.

According to the formula

(1) $$ln\frac{I}{E^{2}}=\ln\left(\frac{A_{eff}\cdot a\beta^{2}}{\emptyset d^{2}}\right)-\frac{b\emptyset^{\frac{3}{2}}}{\beta }s(f)\frac{1}{E}$$

where $A_{eff}$ denotes the effective emission area; a and b are the Fowler–Nordheim (FN) constants, with values of 1.5414 × 10^−6^ and 6.8309 × 10^9^, respectively; d represents the anode-cathode distance; and s(f) is the slope correction function. The work function ∅ is taken as 4.8 eV [19]. The effective emission area and effective field enhancement factor were calculated separately (Table 1).

The typical parameter range of the field enhancement factor for straight looped CNT fibers is approximately 10–100 [20]. Notably, $\beta_{eff}$ is substantially larger than this range, implying that CNT emission tips have formed at the apex. This can be observed in the electron micrograph presented below. Meanwhile, as the heating power increases, the CNTs at the apex may initially fragment, creating additional emission sites. Subsequently, the emission activity stabilizes and localizes onto a limited number of dominant tips, resulting in an initial decrease followed by an increase in the effective field enhancement factor, and a corresponding initial increase followed by a decrease in the field emission area.

In addition, the orthodoxy test [21,22] was employed to validate the thermally assisted electron emission behavior of carbon nanotubes (CNTs). The corresponding formula is given by

(2) $$f^{extr}=\frac{\eta \cdot s(f_{I})}{-S^{fit}{\cdot 1/X}^{extr}}$$

where η denotes the scaling parameter, approximately 9.836 × $\emptyset^{-\frac{1}{2}}$; $s(f_{I})$ is the slope correction function, with an approximate value of 0.95; $S^{fit}$ represents the slope of the MG plot; ${1/X}^{extr}$ refers to the specific value read from the abscissa of the FN plot. The results are shown in Table 2. For ∅ = 5 eV, the condition for orthodox field emission is satisfied when $f^{extr}$ falls within the range of [0.14, 0.43]. Thus, the electron emission of CNTs in the thermally assisted mode is still dominated by field emission.

From the perspective of energy, when no external electric field was applied, a potential barrier of height Φ = E_V_ − E_F_ exists between the Fermi level (E_F_) and the vacuum level (E_V_). At this stage, the electron energy is insufficient to overcome the surface potential barrier, confining electrons within the material. Only electrons with energies higher than the work function Φ can escape from the surface, resulting in negligible electron emissions. Application of a weak external electric field suppresses the surface potential barrier, causing the barrier to deform, lower its maximum, and bend downward. Due to electron quantization, partial electrons gain a finite probability of tunneling through the reduced barrier into the vacuum, resulting in electron emission. As the applied field further increases to the order of 10^8^ V/cm, the barrier height is significantly reduced by ∆Φ and its width is narrowed [23], as shown by E_V_′ in Figure 2c. Heating promotes some electrons to a higher energy state E1 (Figure 2d), from which tunneling into the vacuum occurs more readily [24]. Therefore, thermal assistance enables more facile electron emission from CNT, which consequently lowers the turn-on field and enhances the emission current at a given applied field.

**Figure 3:**
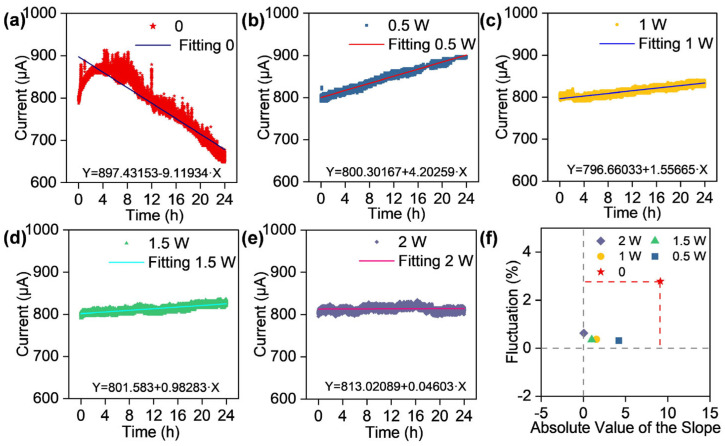
Field emission stability behavior of CNTs under different heating powers. Field emission current–time curves and their fitting functions under different heating powers: (**a**) without heating, (**b**) 0.5 W, (**c**) 1.0 W, (**d**) 1.5 W, (**e**) 2.0 W. (**f**) Scatter plot of fluctuation vs. absolute value of the slope.

To investigate the influence of thermal assistance on the emission electrical stability of CNT cathodes, continuous emission performance tests were conducted on CNT cathodes with heating powers of 0, 0.5, 1, 1.5, and 2 W for a duration of 24 h. Linear fitting of the current–time curves yielded slopes of −9.11934, 4.20259, 1.55665, 0.98283, and 0.04603, respectively (Figure 3a–e). For CNTs without heating, the current exhibits an overall decreasing trend during the 24 h test. In contrast, thermally assisted CNT cathodes exhibit a gradual increase in emission current with time, and higher heating powers result in smaller slopes. The emission current was recorded under constant-voltage conditions. The variation in slope can be regulated via the adjustment of the constant current.

The fluctuation of the emission current was quantified relative to the fitting curve according to the following expression [25]:

(3) $$\begin{array}{c} \begin{array}{c} \begin{array}{c} F_{flu}={\left(\frac{\sum_{i=1}^{n}{\left(I_{i}-I_{i,fit}\right)}^{2}}{n}\right)}^{\frac{1}{2}} \end{array} \end{array} \end{array}\sqrt{I_{fit}}$$

where $F_{flu}$ denotes the fluctuation, $I_{i}$ is the measured current, $I_{i,fit}$ is the fitted current, and $\bar{I_{fit}}$ is the average fitted current. Calculated fluctuation values for heating powers of 0, 0.5, 1.0, 1.5, and 2.0 W are 2.783%, 0.315%, 0.371%, 0.358%, and 0.628%, respectively. Only the unheated CNTs exhibit fluctuation exceeding 1%, while others exhibited fluctuation levels below 1%, with a narrow spread of less than 0.32% among them (between 0.315% and 0.628%).

To quantify the electron emission stability of the CNTs cathode, a plot was constructed with the absolute value of the slope as the abscissa and the corresponding current fluctuation as the ordinate, as presented in Figure 3f. The smaller the product of the distances from a data point to the abscissa zero line and ordinate zero line, the superior the emission stability. The calculated areas for 0 to 2.0 W are 25.379, 1.324, 0.577, 0.352, and 0.028, respectively. These results demonstrate that thermal assistance significantly enhances the field emission stability of CNTs compared with the non-heated condition. Within the range of 0.5–2.0 W, increasing heating power leads to progressively improved stability.

### 3.2. Morphological and Defect Changes Analysis of the Cathode Before and After Field-Emission Testing

**Figure 4:**
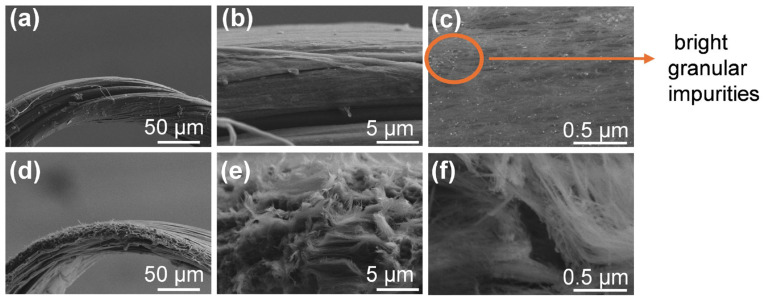
Electron microscopy images of CNTs before and after field emission performance testing: before emission (**a**) 500×, (**b**) 5000×, (**c**) 50,000×; after emission (**d**) 500×, (**e**) 5000×, (**f**) 50,000×.

The morphologies of the CNT surfaces before and after field emission were examined through SEM scans on the V-shaped tip, as illustrated in Figure 4. Prior to testing, the CNT surface contained abundant impurities and exhibited a non-uniform distribution. At 50,000× magnification, the CNTs appeared disorderly arranged with numerous bright granular impurities (Figure 4a–c). After the field emission test, the CNTs showed clear morphological changes. The emission regions were primarily composed of burr-like structures. Internal nanotubes were pulled out, indicating emission-induced consumption. Moreover, high-magnification imaging revealed a notable decrease in impurity particles (Figure 4d–f).

**Figure A3:**
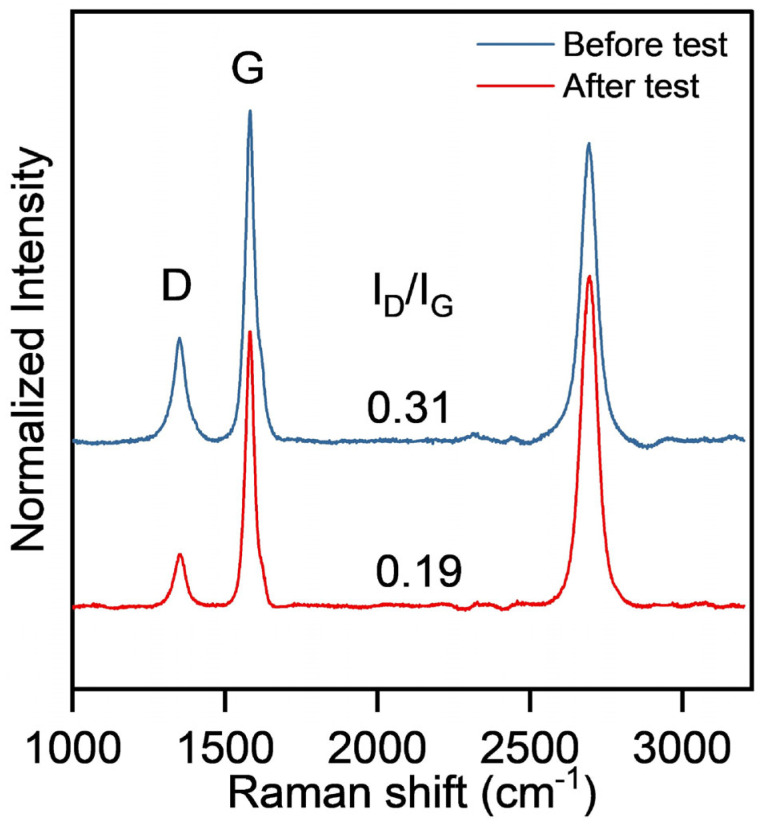
Raman spectra of carbon nanotubes (CNTs) before and after field emission performance testing. D represents the D band, and G represents the G band.

Defect changes in the CNT cold cathodes before and after testing were further characterized through Raman spectroscopy. As shown in Figure A3, two characteristic peaks are observed at approximately 1350 cm^−1^ and 1580 cm^−1^, corresponding to the D-band and G-band, respectively. The D-band is associated with amorphous carbon structures and is commonly used to evaluate defect density [26]. The G-band arises from the E_2_g phonon mode associated with the stretching vibrations of sp^2^-hybridized C-C bonds, serving as a characteristic signature of crystalline carbon [27]. The intensity ratio of the D-band to the G-band (I_D_/I_G_) is a key parameter for quantifying the relative proportions of crystalline and amorphous phases in carbon-based materials [28]. A lower I_D_/I_G_ value signifies a higher degree of structural order, superior crystalline quality, and a reduced density of defects.

Following field emission testing, a decrease in D-band intensity was observed, consistent with the reduction in bright particles in SEM images. This suggests the removal of surface-bound metal catalysts and amorphous carbon, as these defect regions are unstable and are gradually consumed under strong electric fields and current. The I_D_/I_G_ ratio decreased from 0.31 before testing to 0.19 after testing. This indicates that under thermal-assisted conditions, carbon atoms gain sufficient energy to migrate and rearrange, promoting the transformation of amorphous carbon regions into more ordered graphitic structures and improving crystallinity. The defect healing contributes to the enhanced field emission performance of the CNTs.

In addition, gas molecules such as O_2_, H_2_O and CO are commonly adsorbed on the surface of CNTs, forming surface dipole layers and increasing the local work function [29]. Heating promotes the desorption of these adsorbates, which cleans the surface and consequently lowers the work function, thereby improving electron emission performance. After field emission testing, slight morphological reconstruction takes place at the tips, with internal fibers pulled out to form additional fine protrusions. This enhances the local field enhancement factor, as stated in Section 3.1, further lowering the turn-on field and improving emission stability.

### 3.3. Pulsed Electron Emission Performance of CNTs

**Figure A4:**
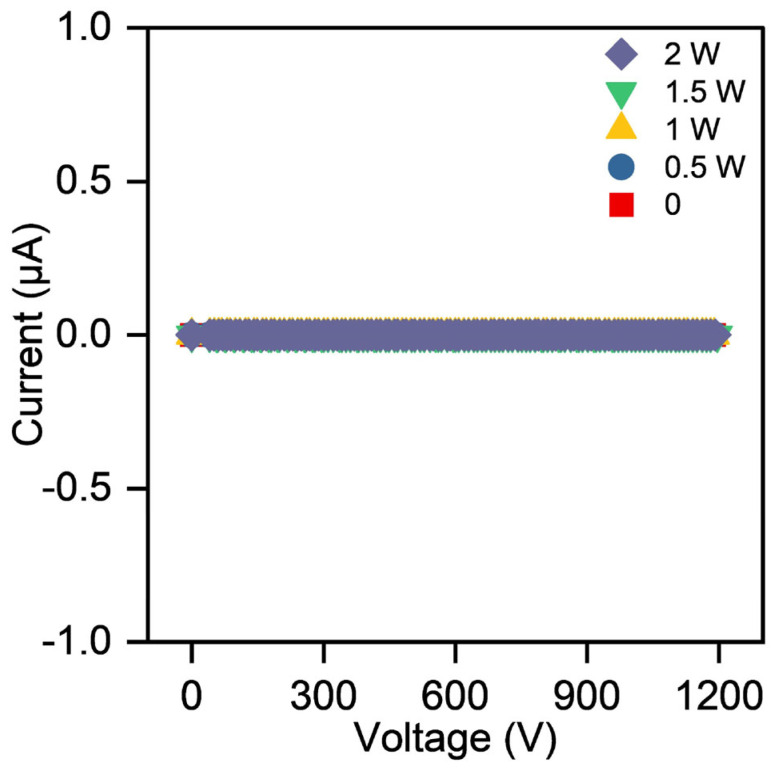
Anode voltage and response current of the response current for pulsed electron emission from CNTs at heating powers ranging from 0 to 2 W.

The pulsed electron emission performance of CNT cold cathodes under thermal assistance was characterized by applying a pulsed positive high voltage to the anode. In this measurement, the overshoot induced by the circuit was suppressed using a smoothing capacitor, which does not affect the evaluation of the response amplitude or rising edge. Figure A4 presents the anode voltage and current responses. When a pulsed voltage of approximately 1200 V was applied to the anode, increasing the heating power from 0 to 2 W resulted in a current increase from 817 μA to 1650 μA. This indicates that at the same anode voltage, the pulsed emission current of CNTs increases with increasing heating power.

**Figure 5:**
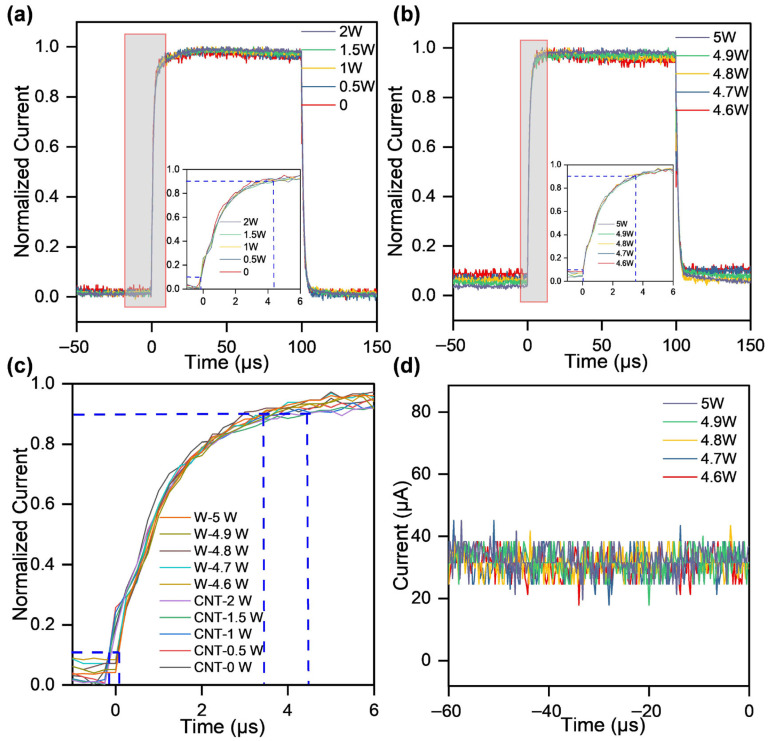
Comparison of thermal-assisted pulsed electron emission from CNTs and tungsten wire. (**a**) Normalized response current and its rising edge of thermal-assisted pulsed electron emission from CNTs with heating power in the range of 0 to 2 W. (**b**) Normalized response current and its rising edge of pulsed electron emission from tungsten wire with heating power ranging from 4.6 to 5 W. (**c**) Comparison of the rising edge of thermal-assisted pulsed electron emission responses between CNTs and tungsten wire. (**d**) The baseline of the response current for pulsed electron emission from tungsten wire.

To carefully analyze the rise time, single pulses were normalized to their peak values, and the duration between 10% and 90% of the peak amplitude was defined as the rise time. Figure 5a shows a comparison of the normalized rise edges. It can be observed that the response speed of CNTs during pulsed electron emission is not affected by heating power, remaining constant at 4.2 μs. These results suggest that thermal assistance primarily promotes electron emission by exciting electrons to higher energy levels, thereby increasing the available emission current, without altering the response speed of the field emission process.

**Figure A5:**
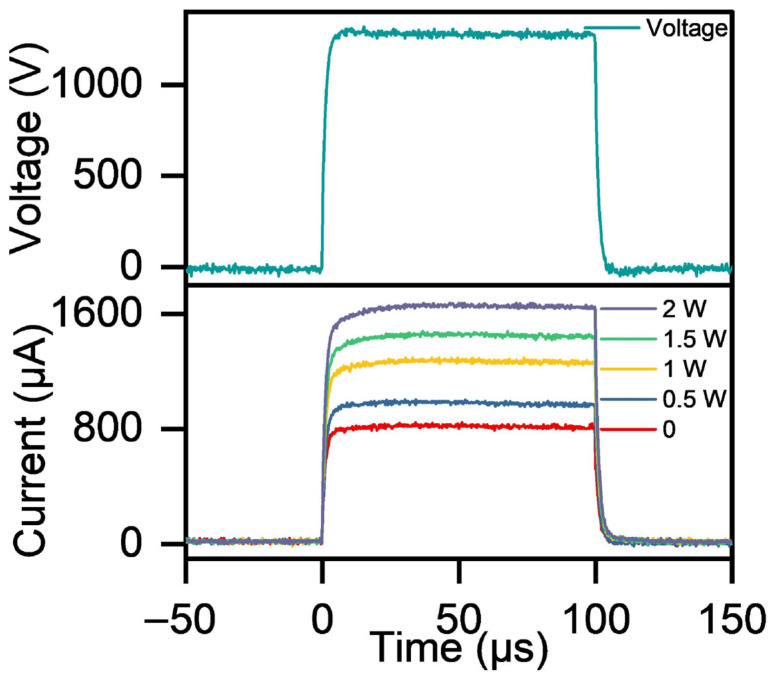
Current vs. anode voltage curves of continuous electron emission from tungsten filaments at heating powers ranging from 0 to 2 W.

To investigate the differences between thermal-assisted electron emission from CNTs and that from metallic materials, tungsten wire was selected as the control group for the experiment. When a DC voltage of 1200 V was applied to the anode, the cathode current remained at 0 within the heating power range of 0–2 W (Figure A5). It demonstrates that the tungsten wire failed to emit electrons under this heating power.

**Figure A6:**
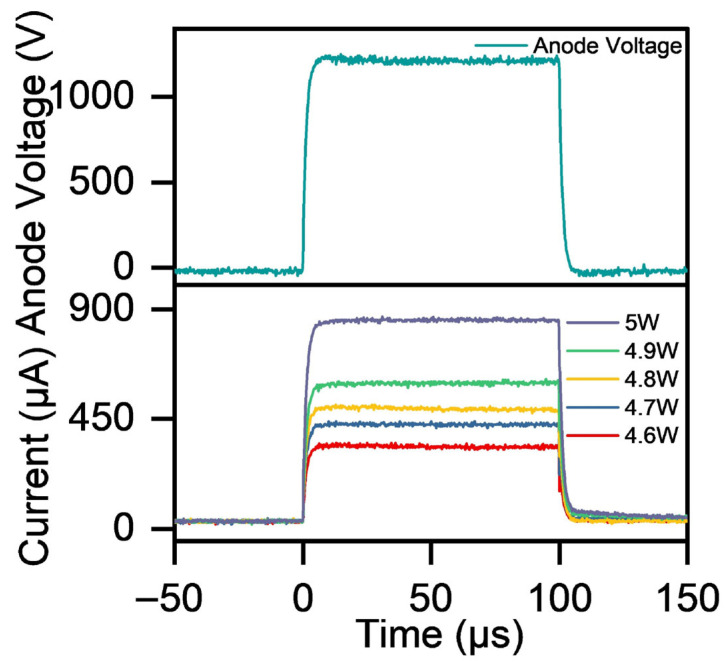
Anode voltage and response current of pulsed electron emission from tungsten filaments at heating powers ranging from 4.6 to 5 W.

The heating power was further increased to 4.6–5.0 W for the tungsten wire, while a positive pulsed high voltage was applied to the anode to monitor the corresponding emission current. When a pulsed voltage of 1200 V was applied to the anode, increasing the heating power from 4.6 W to 5 W resulted in a current increase from 338 μA to 851 μA (Figure A6). It demonstrates a clear positive correlation between the heating power and the pulsed emission current from the tungsten wire. The normalized rising edges are compared in Figure 5b, showing a rise time of 3.7 μs.

From the alignment of normalized pulsed electron emission response rise edges for tungsten wire and CNTs, it is observed that the time required for the current to rise from 10% to 90% of the steady-state value is approximately 4 μs for both materials (Figure 5c). This indicates that both CNTs and tungsten wire can respond to microsecond pulsed voltage sources on the microsecond timescale.

However, due to the more complex microstructure of CNT bundles and the presence of tiny protrusions on their surfaces, which can enhance their local field enhancement factor, CNTs can still generate electron emissions under low electric fields without thermal assistance. With thermal assistance, the CNT cathode requires less heating power. Additionally, the current response of the tungsten wire in pulse mode has a base amplitude of approximately 30 μA (Figure 5d). It indicated that the field emission of tungsten wire under thermal assistance cannot be completely turned off [30]. From the perspective of pulsed imaging, the incomplete turn-off of the tungsten wire’s pulse may increase the artifacts in dynamic imaging and affect its imaging quality. Therefore, CNTs exhibit certain advantages over tungsten wire in terms of electron modulation and energy utilization, offering a reference for the development of novel radiation sources.

### 3.4. Performance Testing and Analysis of X-Ray Generation from CNTs

**Figure 6:**
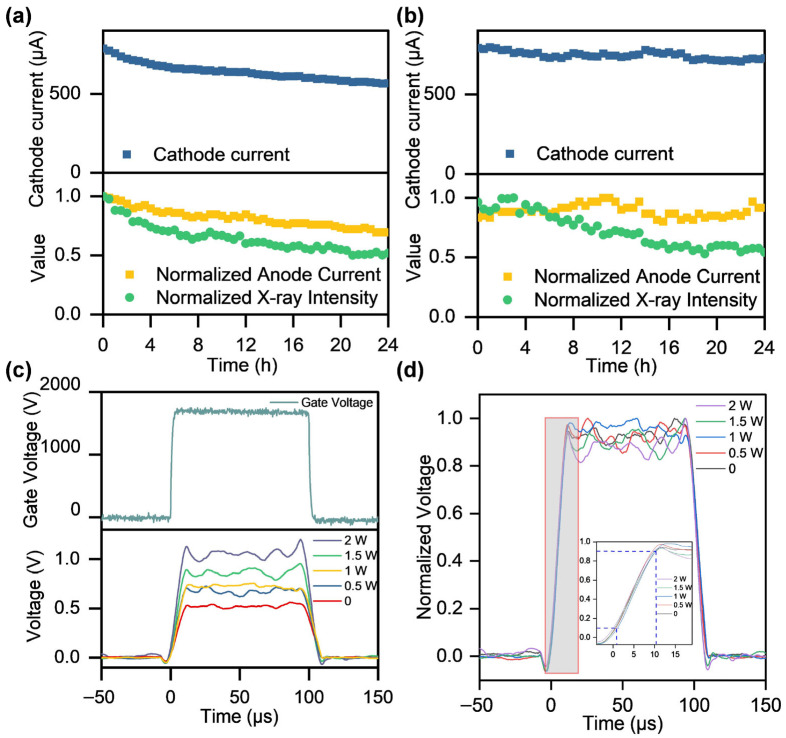
CNT X-ray generation performance in thermal-assisted mode. Curves of anode voltage, normalized gate voltage, and normalized X-ray intensity vs. time at anode powers of (**a**) zero and (**b**) 2 W. (**c**) The gate voltage and X-ray response vs. time for generating pulsed X-rays with CNTs at a heating power of 0–2 W. The X-ray intensity is quantified by the voltage output generated by a photomultiplier tube. (**d**) Normalized responsive X-ray intensity and its corresponding rising edge.

To evaluate the impact of thermal assistance on the X-ray generation performance of CNT cathodes, the most favorable results from continuous electron emission tests were selected for further experimental validation. Figure 6a illustrates that at a heating power of zero, the cathode current decreases from 800 μA to 570 μA, corresponding to a reduction of 28.8%, with an average current value of 650 μA. To facilitate comparison and observation, the anode current and X-ray intensity were normalized. The anode current decreased by approximately 30.4%, with the average normalized anode current being 0.817. The X-ray intensity decreased by about 40.1%, and the normalized X-ray intensity was 0.648.

At a heating power of 2 W, the cathode current decreases only from 800 μA to 700 μA, corresponding to a reduction of 12.5%, with an average cathode current value of 746 μA. The anode current exhibited stable fluctuations, yielding an average normalized anode current of 0.889. The X-ray intensity remained stably fluctuating within the first 4 h, followed by a gradual decrease, and finally exhibited a reduction of 45.7%, with an average normalized X-ray intensity of 0.723 (Figure 6b). Compared with the non-heated case, lower decay rates of the electrical currents and X-ray intensity were measured, with increased average current and X-ray dose.

It should be noted that the cathode current stability in this X-ray experiment was slightly lower than that observed in the continuous two-electrode emission test. This phenomenon is attributed to differences in fabrication processes and structural inconsistencies. The two-electrode test configuration consists solely of a cathode and an anode, where electrons emitted from the cathode are almost entirely collected by the anode, resulting in minimal structural influence. In contrast, the present experiment incorporates a grid electrode with a mesh structure. Additionally, no high-precision cathode centering method was employed, which may have led to uneven electric field distribution and thus the observed reduction in stability [31]. Continuous X-rays can be regarded as the continuous output formed by the tight stacking of countless microscopic pulsed X-rays in time. Thus, it is considered that when a pulse voltage is applied to the grid electrode, the temporal attenuation of the pulsed X-rays generated by the cathode is mitigated by the introduction of thermal assistance, resulting in an increase in the average emitted dose.

The influence of heating power on the pulsed X-ray response was characterized by applying a positive high-voltage pulse to the gate of a CNT cold cathode. The gate voltage is approximately 1680 V, and the X-ray intensity is represented by the voltage signal acquired by the photomultiplier tube circuit. As the heating power increases from 0 to 2 W, the voltage rises from approximately 0.5 V to 1 V (Figure 6c), indicating that thermal assistance enhances the X-ray emission dose. Figure 6d shows the comparison of the normalized X-ray response rise time. The heating power can control the intensity of pulsed X-ray, and the rise time of X-ray can reach 8 μs within the microsecond range. The observed pulse response time is longer than the electron beam rise time due to the temporal broadening inherent in the X-ray generation process via electron-beam-target interactions [32].

**Figure 7:**
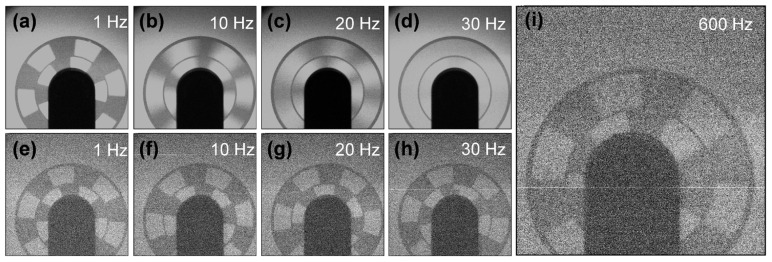
X-ray imaging with thermal-assisted CNTs in continuous and pulsed modes. Continuous X-ray imaging diagrams of fan blades with rotational frequencies of (**a**) 1 Hz, (**b**) 10 Hz, (**c**) 20 Hz, and (**d**) 30 Hz. Pulsed X-ray imaging diagrams of fan blades with rotational frequencies of (**e**) 1 Hz, (**f**) 10 Hz, (**g**) 20 Hz, and (**h**) 30 Hz, with a pulse frequency of 30Hz. (**i**) Pulsed X-ray imaging of fan blades rotating at 600 Hz.

The above experiments demonstrate the applicability and feasibility of thermal-assisted CNT cathodes as X-ray source cathodes. The imaging capability of the X-ray source was evaluated using rotating fan blades as test objects. Imaging was performed with a cathode heating power of 2 W under both direct current and pulsed gate voltage conditions, as shown in Figure 7.

The anode voltage was uniformly set to 45 kV, the pulse voltage frequency was 30 Hz, and the rotational frequencies of the fan blades were 1 Hz, 10 Hz, 20 Hz, and 30 Hz, respectively. At a low frequency of 1 Hz, both continuous and pulsed imaging modes yielded clear visualization of the fan blade shape (Figure 7a,e). When the rotational frequency reached 10 Hz, continuous X-ray imaging exhibited artifacts, with blurred fan blade boundaries, whereas pulsed imaging still maintained clear resolution of the fan blade (Figure 7b,f). At 20 Hz, artifacts in continuous imaging further intensified, yet pulsed imaging retained the ability to resolve the fan blade shape (Figure 7c,g). At 30 Hz, continuous X-ray imaging failed to resolve the blade, and a halo-like region with background-level brightness appeared at its location (Figure 7d). In contrast, the pulsed image retains clear visualization of the blade edges, with no distinct change in brightness (Figure 7h).

To determine the upper limit of imaging speed for pulsed X-ray imaging, the fan blade rotational frequency was raised to its maximum of 600 Hz. The detector still captured clear images of the fan blade, as shown in Figure 7i. These findings demonstrate that the cathode is viable as an electron source cathode for microsecond pulsed imaging, with a temporal resolution significantly superior to that of continuous X-ray imaging. This work thereby provides a reference for the development of advanced pulsed X-ray sources.

## 4. Conclusions

In this article, a V-shaped CNT bundle thermal-assisted field emission electron source was successfully fabricated via the CVD method, which worked with self-Joule heating. Its field-emission properties under thermal-assisted conditions and application for pulsed X-ray imaging were systematically investigated. During continuous electron emission with thermal assistance, the amorphous carbon and metal catalysts on the CNT surface were obviously reduced, the defect density was decreased, and the crystallinity was improved. Thermal assistance lowered the turn-on field by about 0.33 V/μm at maximum and enhanced the stability of electron emission about four times according to current fluctuation. Compared with conventional thermal cathodes (tungsten wire), this cathode exhibits lower power consumption, and its pulse response speed can reach the microsecond scale. The average X-ray dose generated by the CNTs under thermal assistance was 15% higher than no heating, enabling a clear imaging of 600 Hz rotating blades with a 100 μs width pulsed X-ray radiation. This study provides a reference for the development of advanced pulsed X-ray detection technologies in response to the requirements of rapid response and stability for the radiation source in pulsed X-ray imaging.

## Figures and Tables

**Table 1:** Effective field emission area and effective field enhancement factor at different heating powers.

Power	$A_{eff}$(m^2^)	$\beta_{eff}$
0	6.01 × 10^−15^	1922.11
0.5 W	1.97 × 10^−14^	1905.71
1 W	1.20 × 10^−14^	2003.79
1.5 W	6.27 × 10^−15^	2106.31
2 W	2.16 × 10^−15^	2258.63

**Table 2:** Value of f_low_ and f_up_ from the Orthodoxy Test at Different Heating Powers.

Power	f_low_	f_up_
0	0.295	0.398
0.5 W	0.281	0.376
1 W	0.284	0.385
1.5 W	0.292	0.396
2 W	0.300	0.416

## Data Availability

The data presented in this study are available on request from the corresponding authors.

## Abbreviations

The following abbreviations are used in this manuscript:

CNT	Carbon nanotube
AD-MWCNTs	Arc discharge multi-walled carbon nanotubes
DC	Direct current
